# Inhibitory Effects of Trehalose on Malignant Melanoma Cell Growth: Implications for a Novel Topical Anticancer Agent on the Ocular Surface

**DOI:** 10.5402/2012/968493

**Published:** 2012-11-13

**Authors:** Takashi Kudo, Kimio Takeuchi, Yu-ichi Ebina, Mitsuru Nakazawa

**Affiliations:** Department of Opthalmology, Hirosaki University Graduate School of Medicine, 5 Zaifu-cho, Hirosaki 036-8562, Japan

## Abstract

*Purpose*. To investigate the inhibitory effects of trehalose on malignant melanoma cell growth. *Methods*. We cultured human malignant melanoma cells in a medium containing trehalose (control/2.5%/5.0%/7.5%/10.0%) and used the MTT assay to evaluate the growth activities. Subsequently, trehalose was topically instilled on subconjunctivally inoculated melanoma cells in F334/NJcl-rmu/rmu rats, followed by a histopathological evaluation of tumor growth. Using flow cytometry, we compared the distribution of the cell cycle, rate of apoptotic cells, and intracellular factors related to the cell cycle in cultured melanoma cells after trehalose treatment. *Results*. The MTT study showed that proliferation of melanoma cells was significantly inhibited by ≧ 5% of trehalose concentrations in the culture media. Subconjunctivally inoculated melanoma cell masses were significantly smaller in eyes administered trehalose as compared to controls. Flow cytometry analyses demonstrated that the trehalose groups had increased rates of G2/M phase cells and apoptotic cells in the cell culture. These cells also exhibited increased expressions of cell-cycle inhibitory factors. *Conclusions*. The current results show trehalose inhibits malignant melanoma cell growth by inducing G2/M cell cycle arrest and apoptosis, suggesting trehalose as a potential candidate for a topical agent to inhibit proliferation of malignant tumor cells of the ocular surface.

## 1. Introduction

Trehalose, (*α*-glucopyranosyl-(1→1)-*α*-D-glucopyranoside), is a disaccharide isomer of sucrose that has a molecular weight of 342.29 Da with numerous interesting properties. This substance is widely present in animals, plants, insects, and microorganisms and plays an important role in preserving cells in situations that could lead to complete dehydration [1, 2]. Trehalose also reportedly has an inhibitory effect on the denaturation of protein and membranes in bacterial [3] and human cells [4], protects cryopreserved cells under freeze-dried conditions [5] and corneal epithelial cells under dry eye conditions [6], and may potentially have a therapeutic effect when treating the progression of Huntington's disease [7]. The safety and efficacy of topical instillation of trehalose eyedrops have been previously confirmed [8]. In addition to examining the protective effects of trehalose on epithelial cells, we have also previously reported that trehalose exhibited an inhibitory effect on the proliferation of fibroblasts and vascular endothelial cells [9, 10]. Even though trehalose has been shown to have various influences on cell bioactivities as far as we know there have yet to be any investigations that have examined the effects of trehalose on tumor cell growth. Therefore, the current study investigated whether trehalose had inhibitory or protective effects on malignant tumor cells. If trehalose does have an inhibitory effect on tumor cells, this would mean it could potentially be employed as a novel anticancer agent that could be used safely and effectively to topically treat malignant tumors of the ocular surface. 

Various tumors are known to develop in the conjunctiva, with malignant melanoma in particular exhibiting prompt progression. Additionally, since the prognosis for this malignant tumor is one of the worst, the first treatment normally attempted is surgical removal. However, since focal recurrence is frequently seen, a poor prognosis is also usually encountered in cases with hematogenous and/or lymphogenous metastases. In previous studies [9, 10] we demonstrated that trehalose inhibited the proliferation of fibroblasts and vascular endothelial cells by inducing apoptosis. In rabbit glaucoma filtration surgery (GFS) models, we also observed that trehalose inhibited postoperative subconjunctival neovascularization in the filtration bleb areas [9]. Thus, the investigation in the current study specifically focused on whether or not trehalose was able to induce apoptosis of the malignant melanoma cells and, thus, suppress subsequent tumor growth.

## 2. Materials and Methods

### 2.1. Materials

Cell culture dishes, plates, centrifuge tubes, and other plastic ware were purchased from Nunc (Thermo Fisher Scientific, Roskilde, Denmark). We used 10% FBS (Fetal Bovine Serum Life Technologies, Carlsbad, CA, USA) containing DMEM (Dulbecco's Modified Eagle Medium Life Technologies). The human malignant melanoma cell line (MEWO, EC93082609, purchased from European Collection of Cell Cultures, Porton Down, UK) was kindly provided by the Department of Dermatology, Hirosaki University Graduate School of Medicine, Hirosaki, Japan. The 3-[4,5-dimethylthiazol-2-yl]-2,5-diphenyltetrazolium bromide (MTT) cell growth assay kit was purchased from Chemicon International (Temecula, CA, USA). F334/NJcl-rnu/rnu rats, which have genetically lost their T-cell function, were purchased from Clea (Tokyo, Japan). All experimental animal procedures were designed to ethically conform to both the Association for Research on Vision and Ophthalmology Statement for the Use of Animals in Ophthalmic and Vision Research, and to the guidelines of our own institution.

### 2.2. *In Vitro* Study

Effects of trehalose on cultured human melanoma cells were investigated using a previously reported method [9]. Briefly, after human malignant melanoma cells (MEWO) were cultured in a flask, cells were divided in 96 wells, with 5.0 × 10^4^ cells/mL in one cell. The culture media were changed after 24 hours and replaced with media containing various concentrations of trehalose (0%, 2.5%, 5.0%, 7.5%, and 10.0%, resp.). After 3, 6, 8, and 10 days of culture in the trehalose containing media, an MTT test was performed to evaluate the effect of trehalose on melanoma cell growth and survival during *in vitro *culture conditions. The MTT assay measured absorbance and differences in the growth inhibition rates of each cell type at each of the various concentrations of trehalose in the culture media (*n* = 6 in each concentration of trehalose). Results were statistically analyzed using a Kruskal-Wallis test and one-way analysis of variance (ANOVA). Subsequently, statistical differences in each group were tested using a Mann-Whitney *U*-test and Student's* t*-test. Values of *P* < 0.05 were considered to be statistically significant. Statistical analyses were carried out using SPSS version 17 software (Statistical Package for the Social Sciences, Chicago, IL, USA).

### 2.3. *In Vivo* Study

After human malignant melanoma cells were cultured until they became confluent in a T-160 cm flask; 0.1 mL of the melanoma cell suspension (8 × 10^7^ cells/mL of PBS) was injected into the subconjunctival space of the right eye of each of the 5-week-old F334/NJcl-rmu/rmu rats. All injections used a 27-gauge needle and 1.0 mL syringe and were performed under topical anesthesia administered by instillation of 0.4% oxybuprocaine hydrochloride. After the subconjunctival injection, eyedrops containing trehalose (0%, 5.0%, 7.5%, and 10.0% in saline, resp., *n* = 3 each group) were instilled 4 times a day for 28 days. After the instillation period, eyeballs were enucleated followed by evaluation of the tumor size by histopathologic observation. Enucleated eyeballs were fixed overnight in 4% paraformaldehyde solution and treated for 2 h with 10% sucrose in phosphate-buffered saline (PBS), 2 h with 20% sucrose in PBS, and then overnight with 30% sucrose in PBS. After fixation, the eyeballs were embedded in Tissue-Tek (Sakura, Tokyo, Japan) and sectioned in a cryostat using optimum cutting temperature (OCT) compounds for Masson trichrome staining and immunohistochemistry. Frozen fixed eyeballs were dissected along tangential (A) and vertical (B) sections at the center of the tumor. The 4 *μ*m thick sections from the tumor were then immunostained for evaluation of the size of the final tumor mass in each of the groups. Three sections were semiquantitatively observed in each concentration of trehalose.

For immunohistochemistry, tissues were first blocked by 0.5% skim milk in 0.05% Tween 20 in PBS for 1 h at room temperature. The slides were then treated with mouse antihuman melanoma black (HMB) 45 monoclonal antibody (DAKO, Glostrup, Denmark) (1 : 300) as the primary antibody overnight at 4°C. For the second antibody, slides were then treated with Alexa Fluor 568 goat anti-mouse IgG conjugate (Molecular Probes, Eugene, OR, USA) (1 : 200) overnight at 4°C. Nuclei were counterstained with 4′,6′-diamino-2-phenylindole (DAPI). Tissue sections were examined under a fluorescence microscope. Tumor size was measured by using ImageJ software calibrated by a 1.0 mm scale bar. Tumor size differences in each concentration group were analyzed using a Mann-Whitney *U*-test. Values of *P* < 0.05 were considered to be statistically significant.

### 2.4. Flow Cytometry Analysis

We examined the distribution of the cell cycle, rate of apoptosis, and the factors related to the DNA damaged G2 phase cell cycle arrest. These factors which included the ataxia telangiectasia mutated (ATM), p53, check kinase (Chk) 2, and the phosphocell division cycle (p-cdc) 25 in cultured cells by flow cytometry using the antibodies and markers were described below. Briefly, malignant melanoma cells were cultured until they were about 80% confluent, after which the medium was exchanged with media that contained trehalose (0%, 5.0%, and 7.5%, resp.). After 4 days of culture, cells were harvested using a cell scraper and then centrifuged and washed twice with PBS at 1,000 rpm for 5 min at room temperature. Cells were resuspended in 1× binding buffer (BD Biosciences, Franklin Lakes, NJ, USA). After transferring 100 *μ*L of samples to 5 mL culture tubes, BrdU (bromodeoxyuridine) labeled by FITC (fluorescein isothiocyanate) and 7-AAD (7-amino-actinomycin D, BD Biosciences) were added to each of the tubes for the purpose of examination of the cell cycle. The rate of cell death was determined by propidium iodide (PI, BD Biosciences), which detects DNA in dead cells after cell membrane was broken and that of apoptosis was analyzed by annexin V (BD Biosciences), which detects the surface exposure of phosphatidyl serine during apoptosis. Factors related to the DNA damaged G2/M phase cell cycle arrest were examined by using primary antibodies against ATM (rabbit polyclonal IgG against ATM, Abcam, Cambridge, UK), p53 (mouse monoclonal IgG against p53, Abcam), Chk2 (mouse monoclonal IgG against Chk2, Millipore, Billerica, MA, USA), or p-cdc 25 (rabbit polyclonal IgG against p-cdc 25, Abcam) and by using secondary antibodies labeled by either FITC (goat polyclonal IgG against rabbit IgG, Abcam) or PE (phycoerythrin, goat polyclonal F(ab')2 against mouse IgG, Abcam). Cells that positively reacted to the markers and/or antibodies were detected by a fluorescence-activated cell sorting (FACS) flow cytometer (FACSCaliblur, BD Biosciences).

## 3. Results

### 3.1. *In Vitro* Experiments

**Figure 1:**
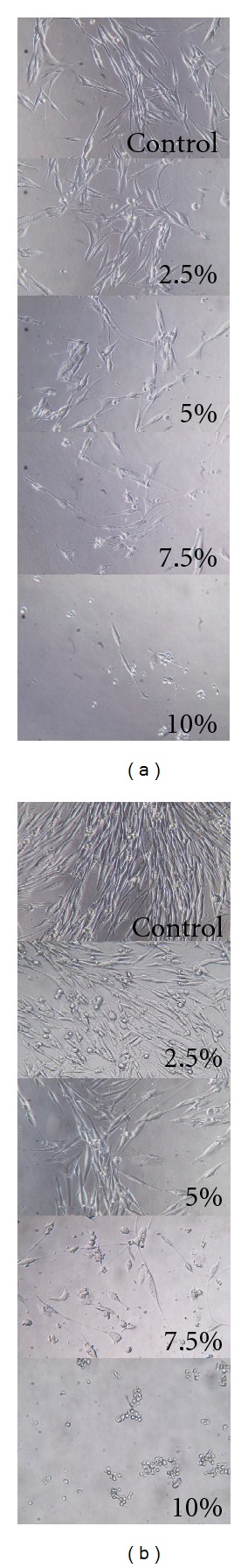
Light microscopic appearances of cultured melanoma cells with or without trehalose (original magnification: ×100). Pictures on the left side present melanoma cells cultured for 3 days, while the right side shows cells cultured for 10 days. Concentrations of trehalose are shown on the lower left corner of each picture. Results show that trehalose inhibited melanoma cell cultures in a dose dependent manner.

**Figure 2:**
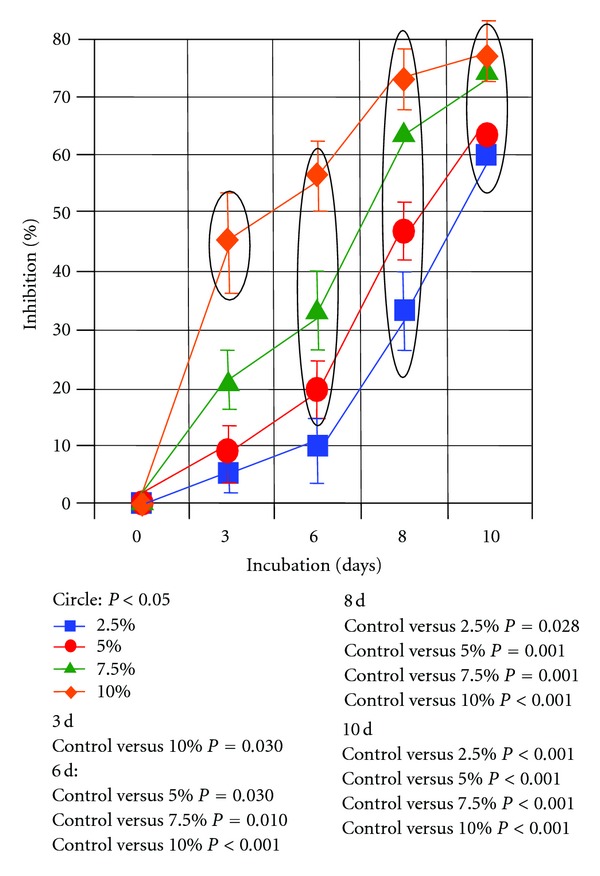
MTT assay results are shown. Circles indicate significant Kruskal-Wallis tests (*P* < 0.05). At 6 days after changing the medium to include trehalose concentrations that were >5%, a significant inhibition in absorbance was noted. Significance levels (*P* values) of differences in cell numbers between trehalose groups and control are indicated in the lower right side corner.

The *in vitro* examinations indicated that rehalose suppressed the growth of malignant melanoma cells. Microscopic observation demonstrated that when higher concentrations of trehalose were present in the culture media, there was a tendency for a stronger suppression of the melanoma cell growth (Figure 1). In addition, the MTT assay measurements showed that the proliferation of melanoma cells was significantly inhibited after 6 days of culture with culture media containing trehalose concentrations of ≧5% (Figure 2). 

### 3.2. *In Vivo* Experiments

**Figure 3:**
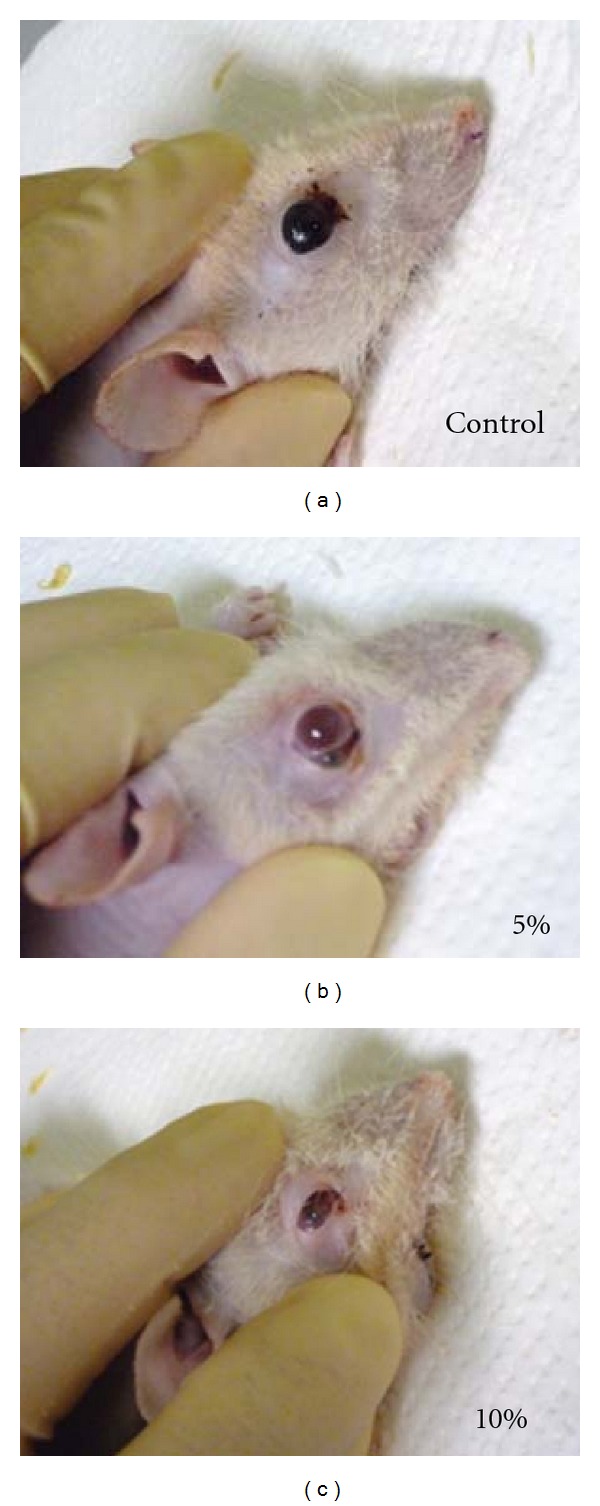
Macroscopic findings for the subconjunctivally inoculated malignant melanoma cells from the F334/NJcl-rmu/rmu rats at 28 days after inoculation. Topical trehalose concentrations were 0% (a), 5% (b), and 10% (c). The tumor masses found in the trehalose instilled groups were macroscopically smaller than those observed in the control group for both the horizontal and vertical dimensions of the tumor.

**Figure 4:**
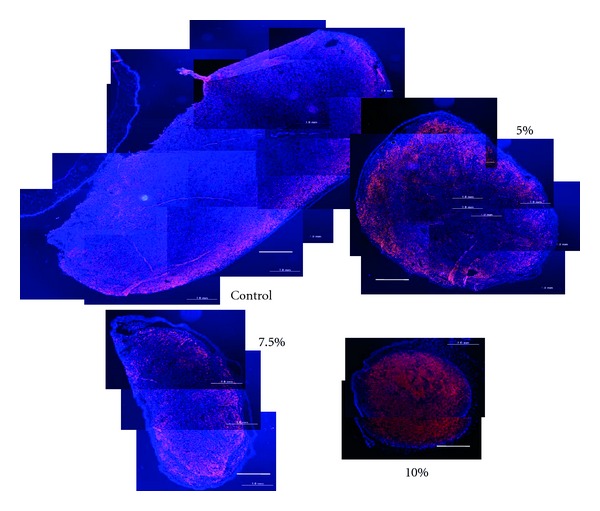
Immunohistochemical findings for the subconjunctivally inoculated malignant melanoma cells. Tissue sections were prepared at the center of the tumor and sizes were compared using the same magnification. Reddish dots represent HMB positive melanoma cells. All photographs were taken at the same magnification, that is, scale bars set at 1.0 mm and ImageJ software was calibrated by the 1.0 mm scale bar.

**Figure 5:**
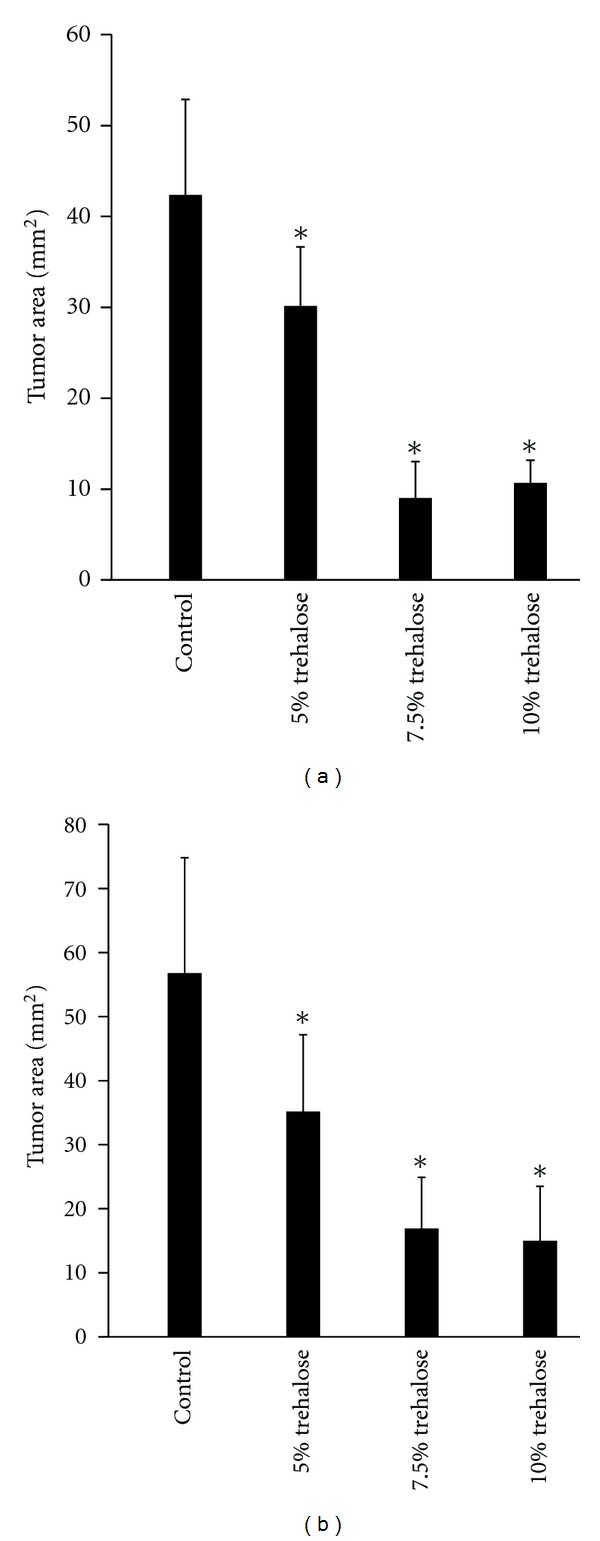
Sizes of the subconjunctivally inoculated melanoma cell 28 days after inoculation treatments with or without trehalose instillation along the tangential (a) and vertical (b) planes at the center of the tumor. Results are presented as the means ± standard deviation are described (*n* = 3). The “∗” denotes significance (*P* < 0.05).

In the *in vivo* examination, inoculated melanoma cell masses treated by trehalose instillation exhibited significantly smaller sizes than the control tumor cells in both the tangential (A) and vertical (B) dimensions of the tumor mass (Figure 3). In the immunohistochemical analysis, the suppressive effects of trehalose on the tumor cell masses showed there was dose dependency for trehalose (Figure 4). The same results were obtained by histopathological analysis using Masson trichrome staining. In addition, the quantitative analysis also indicated that the cross-sections of the tumor areas were significantly smaller in the trehalose-treated groups than in the control group (*P* < 0.05, Figure 5).

### 3.3. Flow Cytometry

**Figure 6:**
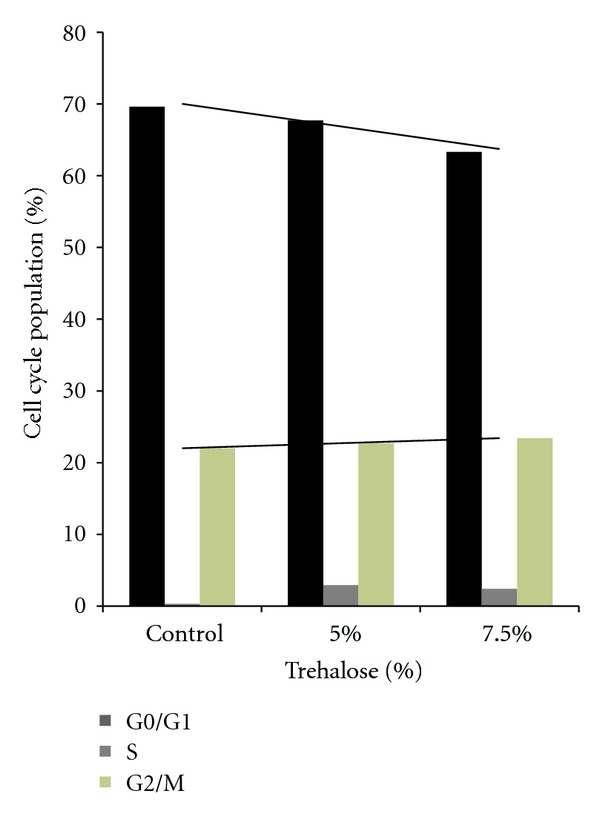
After treatment of the cell cycle populations of the cultured malignant melanoma cells with or without trehalose, a flow cytometry analysis indicated that the trehalose treatment group had a larger number of G2/M phase cells than the controls (PBS).

**Figure 7:**
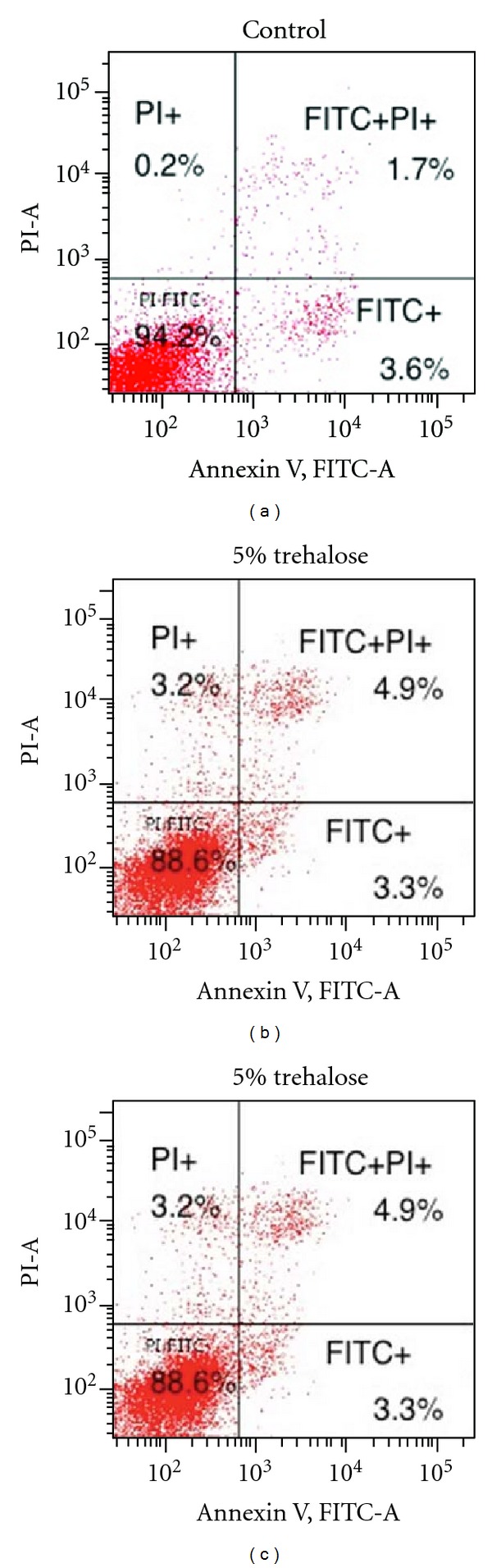
Flow cytometric cell cycle analysis for the melanoma cells with or without trehalose treatment. Cell cycle was analyzed by PI staining and apoptosis was analyzed by annexin V staining. Results indicated that trehalose treatment led to the cell cycle arrest and apoptosis in a dose dependent manner. Data shown are the percentage of cells counted. Abbreviations: PI-A: absorbance for propidium iodide; FITC-A: absorbance for fluorescein isothiocyanate.

**Figure 8:**
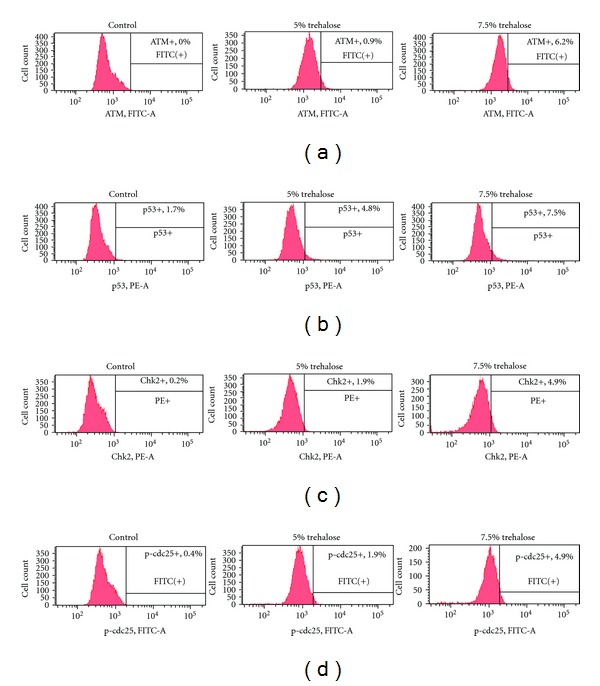
Flow cytometric analyses for the factors in the G2 phase cell cycle. Results for ATM (a), p53 (b), Chk2 (c), and p-cdc25 (d) are shown, respectively, and indicated that trehalose treatment led to increase these factors in a dose dependent manner. Abbreviations: PE-A: absorbance for phycoerythrin; FITC-A: absorbance for fluorescein isothiocyanate.

In the flow cytometry experiments, there was a tendency for the trehalose-treated groups to exhibit a larger number of cells in the G2/M and S phase and a smaller number of G0/G1 phase cells in the cell cycle (Figure 6). Both Figure 7 and Table 1 show the data for the experiments that examined the ratio of the apoptotic cells and cell cycle arrest. These results demonstrate that both PI and annexin V detected larger numbers of apoptotic cells in the trehalose-treated groups versus the control group. In particular, the number of cells positive for both annexin V and PI in the trehalose-treated groups was at least 2.5-times greater than that seen for the control group. These results indicate that apoptotic cell death occurred significantly more frequently in the trehalose-treated group than in the controls. In addition, cells that reacted positively to inhibitory factors in the G2 phase cell cycle, which included ATM, p53, Chk2, and p-cdc25 were detected to a greater in the trehalose-treated groups (2.82–24.5 times) than in the control group (Figure 8 and Table 2).

## 4. Discussion

Trehalose is a widely used ingredient in foods, cosmetics, and medicine, as it is considered to be safe for human consumption. In the field of ophthalmology, trehalose has been suggested as a possible topical agent that can be used to effectively treat dry eye [6] and as a preservative agent to keep amniotic membranes viable under freezing conditions [5]. In our previous studies [9, 10], we demonstrated that trehalose exhibited no adverse effects for either the normal conjunctiva or corneal epithelium. Moreover, it is also reported that trehalose can be safely used as a topical agent [8].

We previously demonstrated that trehalose prevented fibroblasts from changing into myofibroblasts during the process of postoperative subconjunctival scar formation after a simple incision of the conjunctiva or after surgical invasion such as a trabeculectomy [9]. These experiments led us to speculate that trehalose eyedrops might inhibit the postoperative cicatrizations that occur after eye surgery. Although antimetabolic agents such as 5-fluorouracil and mitomycin C are widely used to control postoperative subconjunctival fibrous scar formation after GFS like trabeculectomy [11, 12], the effects of these antimetabolites tend to be irreversible and can cause serious blinding complications such as avascular filtration bleb, conjunctival button-hole formation, and infection of the filtering bleb that can subsequently lead to endophthalmitis during the later stages [13–18]. During the healing process for the GFS wound, we previously found that trehalose was able to inhibit both the VEGF-induced neovascularization and proliferation of myofibroblasts by increasing the apoptosis of fibroblasts [10]. We also determined that since the osmotic pressures for 5% trehalose and saline solution were equivalent, 5% trehalose caused no osmotic damage to the cells. However, we also noted the possibility that other mechanisms could be responsible for inhibiting the fibroblast proliferation and inducing the apoptosis. The important points of our previous studies were that (1) trehalose inhibits the proliferation of fibroblasts and newly formed vascular endothelial cells, and (2) trehalose has no harmful effects on either conjunctival or corneal epithelial cells. Based on these previous speculations, we decided to use malignant melanoma cells to determine whether or not trehalose has a similar growth inhibitory effect on neoplastic cells. In addition, we also tried to elucidate the molecular mechanisms associated with the trehalose-caused inhibition of the proliferating cells. 

Our current results suggested that another novel characteristic of trehalose might be the prevention of malignant melanoma cells proliferation both* in vitro* (Figures 1 and 2) and *in vivo *(Figures 3, 4, and 5). Flow cytometric analyses also clearly demonstrated that trehalose induced cell death by apoptosis in the tumor cells detected by annexin V and PI (Figure 7). This is the first study that has examined both the expression of the cell cycle regulatory factors and whether or not trehalose affects the cell cycles. The present results demonstrated that ATM, p53, Chk2, and p-cdc25, which were factors that all of the cells were positive for, apparently increased in the trehalose-treated melanoma cells *in vitro *(Figure 8).

ATM is a serine/threonine protein kinase that activates checkpoint signaling factors such as p53 and Chk2 after DNA damage and leads to subsequent apoptosis, thereby acting as a DNA damage sensor and regulating DNA damage response mechanism [19, 20]. In many tumor types, p53 acts as a tumor suppressor and induces growth arrest or apoptosis depending on the physiological circumstances and the cell types [21]. In addition, p53 is involved in cell cycle regulation as a transactivator, which acts to negatively regulate cell division by controlling a set of genes involved in the regulation of this process [20]. Apoptosis induction appears to be mediated either by stimulation of BAX and FAS antigen expression, or by the repression of Bcl-2 expression [22]. Chk2 regulates cell cycle checkpoints and apoptosis in response to DNA damage, particularly to DNA double-strand breaks. It also inhibits cdc25c phosphatase by phosphorylation and subsequently prevents the entry into mitosis [23]. The p-cdc25 is a phosphorylated and inactivated form of cdc25 that dephosphorylates cyclin B1/cdc2, which is essential for the progression through the G2/M phase of the cell cycle [24]. Thus, increased amounts of p-cdc25 lead to inhibition of the progression into mitosis [25, 26]. 

**Figure 9:**
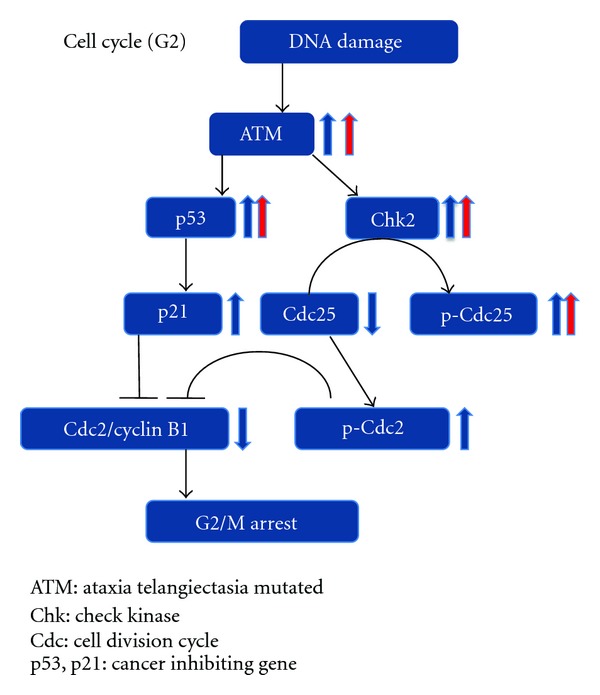
Relationships between the factors in the G2 phase cell cycle. As indicated by the blue arrows, both increases (ATM, p53, Chk2, p21, p-cdc25) and decreases (cdc25 and cdc2/cyclin B1) of factors lead to the arrest of the G2/M cell cycle (blue arrows). After treatment with trealose, an increase was seen in the ATM, p53, Chk2, and p-cdc25 factors (red arrows). These results suggest the possibility that administration of trehalose leads to the G2/M cell cycle arrest and to apoptosis.

 As summarized and speculated in Figure 9, ATM, p53, Chk2, and p-cdc25 are some of the key factors that inhibit the progression to mitosis (G2/M arrest), thereby inducing apoptosis and/or the arrest of proliferation. The current results demonstrated that trehalose treatment increased the protein levels of these factors. Although the exact molecular mechanisms responsible for growth inhibition are still unknown, our current findings suggest the possibility that trehalose may be able to inhibit the proliferation of melanoma cells by probably inducing apoptosis and/or G2/M arrest.

From a clinical standpoint, the current results may have implications with regard to the development of a novel topical application of trehalose as eyedrops for inhibiting malignant neoplasms that occur on the ocular surface region. In the future, this treatment may provide a way to safely inhibit the proliferation of tumor cells grown on the surface of the eye.

Although the present study specifically focused on malignant melanoma, pterygium, and other diseases involving neoplastic cells such as conjunctival squamous cell carcinoma are clinically more common. Therefore, we are in the process of pursuing further studies that will examine whether or not trehalose eyedrops can similarly inhibit proliferation in these types of diseases.

## Figures and Tables

**Table 1:** Summary of flow cytometric analysis for ratios of annexin V positive and PI positive cells in various concentrations of trehalose in culture media shown in Figure 7.

	Annexin V+ (total)	Annexin V+/PI+
	Apoptotic cells	Cell cycle arrest
Control	5.30%	1.70%
5% trehalose	8.20%	4.90%
7.5% trehalose	13.20%	5.4%

**Table 2:** Summary of flow cytometric results for ratios of ATM, P53, Chk2, or p-cdc25 positive cells in various concentrations of trehalose in culture media in Figure 8.

	ATM+	P53+	Chk2+	p-cdc25+
Control	0.0%	1.7%	0.2%	0.4%
5% trehalose	0.9%	4.8%	1.9%	1.9%
7.5% trehalose	6.2%	7.5%	4.9%	4.9%

Abbreviations: ATM: ataxia telangiectasia mutated; Chk: check kinase; p-cdc: phosphorylated cell division cycle.

## References

[1] Crowe JH, Hoekstra FA, Crowe LM (1992). Anhydrobiosis. *Annual Review of Physiology*.

[2] Mayer RJ, Candy DJ (1969). Changes in energy reserves during flight of the desert locust, schistocerca gregaria. *Comparative Biochemistry And Physiology*.

[3] Leslie SB, Israeli E, Lighthart B, Crowe JH, Crowe LM (1995). Trehalose and sucrose protect both membranes and proteins in intact bacteria during drying. *Applied and Environmental Microbiology*.

[4] Guo N, Puhlev I, Brown DR, Mansbridge J, Levine F (2000). Trehalose expression confers desiccation tolerance on human cells. *Nature Biotechnology*.

[5] Nakamura T, Sekiyama E, Takaoka M (2008). The use of trehalose-treated freeze-dried amniotic membrane for ocular surface reconstruction. *Biomaterials*.

[6] Matsuo T, Tsuchida Y, Morimoto N (2002). Trehalose eye drops in the treatment of dry eye syndrome. *Ophthalmology*.

[7] Tanaka M, Machida Y, Niu S (2004). Trehalose alleviates polyglutamine-mediated pathology in a mouse model of Huntington disease. *Nature Medicine*.

[8] Matsuo T (2004). Trehalose versus hyaluronan or cellulose in eyedrops for the treatment of dry eye. *Japanese Journal of Ophthalmology*.

[9] Takeuchi K, Nakazawa M, Ebina Y (2010). Inhibitory effects of trehalose on fibroblast proliferation and implications for ocular surgery. *Experimental Eye Research*.

[10] Takeuchi K, Nakazawa M, Ebina Y (2011). Effects of trehalose on VEGF-stimulated angiogenesis and myofibroblast proliferation: implications for glaucoma filtration surgery. *Investigative Ophthalmology and Visual Science*.

[11] Kitazawa Y, Kawase K, Matsushita H, Minobe M (1991). Trabeculectomy with mitomycin: a comparative study with fluorouracil. *Archives of Ophthalmology*.

[12] Kitazawa Y, Suemori-Matsushita H, Yamamoto T, Kawase K (1993). Low-dose and high-dose mitomycin trabeculectomy as an initial surgery in primary open-angle glaucoma. *Ophthalmology*.

[13] Sihota R, Dada T, Gupta SD, Sharma S, Arora R, Agarwal HC (2000). Conjunctival dysfunction and mitomycin C-induced hypotony. *Journal of Glaucoma*.

[14] Solomon A, Ticho U, Frucht-Pery J (1999). Late-onset, bleb-associated endophthalmitis following glaucoma filtering surgery with or without antifibrotic agents. *Journal of Ocular Pharmacology and Therapeutics*.

[15] Poulsen EJ, Rand Allingham R (2000). Characteristics and risk factors of infections after glaucoma filtering surgery. *Journal of Glaucoma*.

[16] Greenfield DS, Parrish RK (1996). Bleb rupture following filtering surgery with mitomycin-C: clinicopathologic correlations. *Ophthalmic Surgery and Lasers*.

[17] Belyea DA, Dan JA, Stamper RL, Lieberman MF, Spencer WH (1997). Late onset of sequential multifocal bleb leaks after glaucoma filtration surgery with 5-fluorouracil and mitomycin C. *American Journal of Ophthalmology*.

[18] DeBry PW, Perkins TW, Heatley G, Kaufman P, Brumback LC (2002). Incidence of late-onset bleb-related complications following trabeculectomy with mitomycin. *Archives of Ophthalmology*.

[19] Bouchet BP, Caron de Fronmentel C, Puisleux A, Galmarini CM (2006). P53 as a target of anti-cancer drug development. *Critical Reviews in Oncology/Hematology*.

[20] Stracker TH, Usui T, Petrini JHJ (2009). Taking the time to make important decisions: the checkpoint effector kinases Chk1 and Chk2 and the DNA damage response. *DNA Repair*.

[21] Shangary S, Qin D, McEachern D (2008). Temporal activation of p53 by a specific MDM2 inhibitor is selectively toxic to tumors and leads to complete tumor growth inhibition. *Proceedings of the National Academy of Sciences of the United States of America*.

[22] Fridman JS, Lowe SW (2003). Control of apoptosis by p53. *Oncogene*.

[23] Reinhardt HC, Yaffe MB (2009). Kinases that control the cell cycle in response to DNA damage: Chk1, Chk2, and MK2. *Current Opinion in Cell Biology*.

[24] Uhlmann F, Bouchoux C, López-Avilés S (2011). A quantitative model for cyclin-dependent kinase control of the cell cycle: revisited. *Philosophical Transactions of the Royal Society B*.

[25] Baldi A, DeLuca A, Esposito V, Camprioni M, Spugnini EP, Citro G (2011). Tumor suppressors and cell-cycle proteins in lung cancer. *Pathology Research International*.

[26] Strausfeld U, Fernandez A, Capony JP (1994). Activation of p34(cdc2) protein kinase by microinjection of human cdc25C into mammalian cells. Requirement for prior phosphorylation of cdc25C by p34(cdc2) on sites phosphorylated at mitosis. *The Journal of Biological Chemistry*.

